# Improved Pyroelectric Nanogenerator Performance of P(VDF-TrFE)/rGO Thin Film by Optimized rGO Reduction

**DOI:** 10.3390/nano14221777

**Published:** 2024-11-05

**Authors:** Hafiz Muhammad Abid Yaseen, Sangkwon Park

**Affiliations:** Department of Chemical and Biochemical Engineering, Dongguk University, 30 Pildong-ro 1-gil, Jung-gu, Seoul 04620, Republic of Korea; engineerabid786@yahoo.com

**Keywords:** pyroelectric nanogenerator (PyNG), P(VDF-TrFE)/rGO nanocomposite, thin film, rGO reduction, energy harvesting performance

## Abstract

The pyroelectric nanogenerator (PyNG) has gained increasing attention due to its capability of converting ambient or waste thermal energy into electrical energy. In recent years, nanocomposite films of poly(vinylidene fluoride-co-trifluoro ethylene) (P(VDF-TrFE)) and nanofillers such as reduced graphene oxide (rGO) have been employed due to their high flexibility, good dielectric properties, and high charge mobility for the application of wearable devices. This work investigated the effect of rGO reduction on pyroelectric nanogenerator performance. To prepare rGO, GO was reduced with different reducing agents at various conditions. The resulting rGO samples were characterized by XPS, FT-IR, XRD, and electrical conductivity measurements to obtain quantitative and qualitative information on the change in surface functionalities. Molecularly thin nanocomposite films of P(VDF-TrFE)/rGO were deposited on an ITO-glass substrate by the Langmuir–Schaefer (LS) technique. A PyNG sandwich-like structure was fabricated by arranging the thin films facing each other, and it was subjected to the pyroelectric current test. For various PyNGs of the thin films containing rGO prepared by different methods, the average pyroelectric peak-to-peak current (APC) and the pyroelectric coefficient (*p*) values were measured. It was found that a more reduced rGO resulted in higher electrical conductivity, and the thin films containing rGO of higher conductivity yielded higher APC and *p* values and, thus, better energy-harvesting performance. However, the thin films having rGO of too high conductivity produced slightly reduced performance. The Maxwell–Wagner effect in the two-phase system successfully explained these optimization results. In addition, the APC and *p* values for the thin film with the best performance increased with increasing temperature range. The current PyNG’s performance with an energy density of 3.85 mW/cm^2^ and a *p* value of 334 μC/(m^2^∙K) for ΔT = 20 °C was found to be superior to that reported in other studies in the literature. Since the present PyNG showed excellent performance, it is expected to be promising for the application to microelectronics including wearable devices.

## 1. Introduction

In recent years, pyroelectric energy harvesting has gained increasing attention due to its capability to convert ambient or waste thermal energy, such as the heat generated by the human body, vehicle exhausts, the industrial cooling process, and solar radiation, into electrical energy [1,2]. This energy conversion technology can be a good approach to obtain renewable energy and power sources for low-power portable electronics, self-powered sensors, and other wearable devices without external power sources like batteries [2,3,4]. Although a conventional thermoelectric generator can be used to convert waste heat energy into electrical energy, it is based on spatial temperature gradients (*dT/dx*) between two different thermoelectric materials (Seebeck effect). Meanwhile, the pyroelectric nanogenerator (PyNG) is based on temperature fluctuations (*dT/dt*), and thus, it is more effective compared to the thermoelectric method [1,4].

Pyroelectricity happens when the polarization of electrically polarized material is varied due to temperature fluctuation. In some materials like ferroelectrics, there are opposite charges at both ends, and under thermal equilibrium, these charges are equal without a net charge. However, when the temperature fluctuates, the material’s polarization will change in response to the temperature fluctuations, and thus, corresponding electric fields are generated [1]. The most commonly used pyroelectric materials can be classified into several different types: ferroelectric ceramics, such as PZT, BaTiO_3_, and PbTiO_3_; ferroelectric organic–inorganic crystals, such as triglycine sulfate; inorganic crystals, such as LiTaO_3_; and organic crystals, like amantadine formate, ferroelectric polymers such as poly(vinylidene fluoride) (PVDF) and poly(vinylidene fluoride-co-trifluoro ethylene) (P(VDF-TrFE)), and non-ferroelectric crystals such as CdS and ZnO [1,2,5]. The pyroelectric coefficient, *p*, defined as the spontaneous polarization (P_S_) change per unit temperature change, i.e., *dP_S_/dT*, is used to characterize the pyroelectric effect of a material. In general, the *p* values of ferroelectric materials are larger than those of non-ferroelectric ones, and those of ceramics and crystals are larger than those of polymers [1]. The ferroelectric semi-crystalline polymer PVDF and P(VDF-TrFE) have been widely employed in pyroelectric nanogenerators (PyNGs) for wearable devices mainly due to high flexibility as well as good stability, high Curie temperature, and chemical and high-temperature resistance [3]. However, the application of PVDF and its copolymers has been limited due to their low dielectric properties and poor charge mobility [6]. To overcome these limitations, many researchers have tried to employ different kinds of nanofillers in the polymer matrix. Typical examples of the most commonly used nanofillers are graphene oxide (GO) and reduced graphene oxide (rGO) to take advantage of their high thermal and electrical conductivities, high surface area improving dispersion and compatibility by allowing for more interaction with the polymer matrix, and ability to improve the dielectric properties of the matrix polymer [7,8,9]. Importantly, rGO has been reported to increase the content of critical β-phases in the polymer matrix [8,9].

Graphene oxide (GO) contains different kinds of oxygen functional groups (OFGs), such as hydroxyl, epoxy, carbonyl, and carboxyl groups, which make the GO surface hydrophilic and less conductive. The hydrophilic nature of GO limits its compatibility with non-polar polymers such as P(VDF-TrFE), making it difficult to form a homogeneous blend with the polymer matrix [10]. In addition, the presence of OFGs at the GO surface can act as defects, leading to a lower thermal and electrical conductivity of the composite film [11,12]. Reduction of GO to rGO by removing OFGs not only results in a more conductive surface by restoring the conjugated sp^2^ carbon network of graphene but also allows for compatibility with the non-polar polymer matrix by making it hydrophobic. In addition, the reduction process removes defect sites, enhancing the thermal and electrical conductivity of the rGO nanofillers [11,12]. Recently, various methods have been used to prepare rGO from GO, and they can be categorized into four methods: chemical reduction, thermal reduction, electrochemical reduction, and microwave reduction [13,14,15,16,17,18,19,20,21,22,23,24,25,26,27,28]. Chemical reduction uses either toxic reducing agents, such as hydrazine monohydrate [14], hydroquinone [15], hydroxylamine [16], sodium borohydride [17,18], and hydrohalic acid [12,18,19], or eco-friendly reducing agents, such as L-ascorbic acid (AA) [20,21], alcohols [22], and amino acids [23]. In thermal reduction, OFGs are removed in the form of water, carbon monoxide, and carbon dioxide by thermal annealing [13,24]. Electrochemical reduction employs an electrolyzing process to reduce GO; this method is fast, and rGO can be prepared with less contaminants [13,25]. In the microwave method, microwave irradiation is usually used to deoxygenate GO surfaces [13,26,27]. For the chemical method, most of the reducing agents are strong, but they are highly toxic, and thus, their use must be minimized to protect the environment; whereas, some eco-friendly reducing agents are not harmful to the environment, but their reduction power is known to be too mild to highly reduce GO [13,20,21,22,23].

In general, the pyroelectric coefficient, *p*, of a PyNG is known to be significantly influenced by the electrical and dielectric properties of pyroelectric materials [10,28]. Therefore, the pyroelectric performance of a PyNG comprising a ferroelectric polymer (PVDF and copolymers) film matrix and rGO is expected to be closely related to the degree of reduction of rGO. However, there has not been any systematic study in this regard, even though some studies have reported that reduction may enhance the electrical conductivity of rGO [11,12,13,29,30].

In this work, for the first time to our knowledge, we investigated the effect of the rGO preparation method on the energy-harvesting performance of a PyNG comprising a P(VDF-TrFE)/rGO nanocomposite thin film. To prepare the nanocomposite film, GO samples were reduced with both strong chemical-reducing agents, HI, NaBH_4_, and hydrazine hydrate, and a mild but eco-friendly reducing agent, L-ascorbic acid (AA). Thin nanocomposite films containing the rGO in the P(VDF-TrFE) matrix were then deposited on conductive ITO-glass substrates by the Langmuir–Schaefer (LS) technique, which is a good method of depositing uniform, highly ordered molecularly thin films on solid substrates [31,32]. The thin film, rather than a thick film or fiber-based film, was employed because the thinner film of rGO was reported to give higher electrical conductivity due to its higher structural order and shorter path length for electron flow [33]. A PyNG of the sandwich-like structure was assembled to make two thin films that faced each other, covered with a thermal pad, connected to electrodes, and located at the center of an automatic Peltier system. The PyNGs were subjected to pyroelectric current measurements in three different temperature ranges to evaluate the average peak-to-peak pyroelectric current (APC) and the pyroelectric coefficient (*p*) values. The APC and *p* values for various PyNGs containing rGO samples of different magnitudes of reduction were measured, and the results were compared with those reported in the literature.

## 2. Materials and Methods

### 2.1. Materials

P(VDF-TrFE) (70:30 mol%), sodium hydroxide (NaOH, 98%), acetone (99.5%), *N*,*N*-dimethylformamide (DMF, 99.8%), ethanol (96%), indium tin oxide coated glass (ITO-glass), natural graphite (Gr, carbon content 88%), potassium permanganate (KMnO_4_, ≥99%), sodium nitrate (NaNO_3_, 70%), sulfuric acid (H_2_SO_4_, 98%), hydrochloric acid (HCl, 37%), hydrogen peroxide (H_2_O_2_, 30%), L-ascorbic acid (AA, reagent grade), sodium borohydride (NaBH_4_, 98%) hydrazine hydrate (N_2_H_4_, 80%), and hydroiodic acid (HI, 55%) were purchased from Sigma-Aldrich, St. Louis, MO, USA, and they were used without any further purification. Deionized (DI) water with a resistivity of 18.3 MΩ·cm was used.

### 2.2. Preparation of GO

GO samples were prepared by the Hummers’ method, according to the protocol reported in the literature [9]. In brief, a mixture of 3.0 g of natural graphite and 1.5 g of NaNO_3_ was added to 69 mL of H_2_SO_4_, and the mixture was stirred during cooling with an ice-water bath. While the mixture was continuously stirred, 9.0 g of KMnO_4_ was added to the mixture, ensuring that the temperature remained below 10 °C, and it was then maintained at 37 °C for 2 h. At an elevated temperature of 96 °C, 138 mL of deionized water was added dropwise to the mixture, and the mixture was stirred for 30 min, followed by the addition of 420 mL of deionized water and 30 mL of an aqueous solution of H_2_O_2_ to remove the residual KMnO_4_. The mixture was then washed with 5% HCl aqueous solution and deionized water and centrifuged at 2500 rpm for 30 min. The precipitated rGO particles were washed to remove SO_4_^2−^ and dried at 60 °C for 24 h under vacuum.

### 2.3. Preparation of rGO

First, the GO samples were reduced by HI according to the procedure in the literature [12]. A total of 160 mg GO was dispersed in 200 mL of DI water by ultrasonication for 5 min in a Teflon beaker, and a specific amount of 55% HI (2.32, 4.64, 11.6, or 23.2 g) was added to the GO dispersion. The mixture was heated at 90 °C in a water bath and kept at constant temperature under vigorous stirring for 4 h. The resulting mixture was cooled down to ambient temperature. The rGO particles were thoroughly washed with DI water and ethanol alternatively many times, and the rGO sample was dried under a vacuum overnight at 60 °C. The samples treated with 2.32, 4.64, 11.6, and 23.2 g HI were denoted as rGO-HI-1, rGO-HI-2, rGO-HI-3, and rGO-HI-4, respectively.

Second, the GO samples were reduced by AA according to the procedure in the literature [21], i.e., 100 mg of AA was added to a 100 mL aqueous GO dispersion with the GO concentration of 0.1 mg mL^−1^ under stirring at 65 °C for different times: 2 h, 1 day, 2 days, and 3 days. The rGO samples prepared by the AA reduction for different times were labeled as rGO-AA-2h, rGO-AA-1d, rGO-AA-2d, and rGO-AA-3d, respectively.

Third, as a reference, an rGO sample was prepared by NaBH_4_ reduction according to the typical method in the literature [17]. In the method, NaBH_4_ (2.28 g) was added to an aqueous suspension of GO (200 mL), the mixture was continuously stirred at room temperature for 12 h, and the resulting rGO sample was subjected to multiple washing with DI water. This rGO sample was denoted as rGO-SBH. As another reference, a GO sample was also prepared by hydrazine hydrate reduction according to the protocol reported in the literature [14]. In this protocol, 1 mL of hydrazine hydrate was added to 100 mL of an aqueous GO suspension (1 mg/mL), and the mixture was subsequently heated with a water-cooled condenser at 100 °C for 24 h. The resulting rGO sample was then subjected to washing alternatively with DI water and methanol, followed by drying at 50 °C under vacuum. This rGO sample was denoted as rGO-Hyd.

### 2.4. Preparation of Nanocomposite Solution and Thin Film

**Figure 1:**
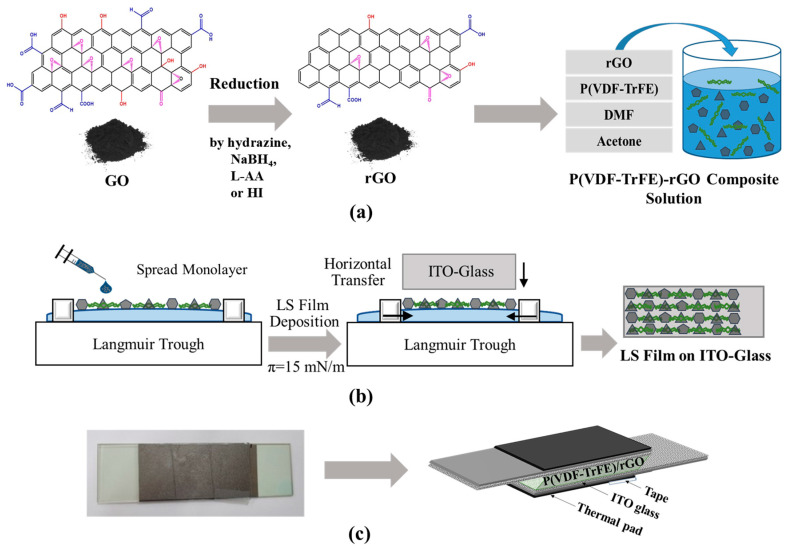
Illustration of processes for (**a**) reduction of GO and (**b**) LS thin film preparation, and (**c**) picture of actual PyNG and its detailed structure.

Eleven solutions of P(VDF-TrFE)/GO and nanocomposite were prepared by dissolving the polymer in DMF/acetone (*v*/*v* 40:60) at the concentration of 0.05 wt%, dispersing different GO and/or rGO samples in the polymer solution at the concentration of 4 wt%, and stirring the mixtures for 12 h. To obtain the P(VDF-TrFE) nanocomposite thin film, the Langmuir–Schafer (LS) technique was used. A Langmuir trough was filled with DI water, and the nanocomposite solution was spread at the air/water interface by a micro-syringe. The nanocomposite monolayer was compressed to a target surface pressure (5 mN/m) by two barriers and transferred to the ITO-glass substrate by touching the monolayer-spread surface with the substrate horizontally. An LS thin film of multilayers was prepared by repeating the monolayer deposition procedure ten times. Finally, the thin film was dried in the air at room temperature and stored in a desiccator for further analysis and experiments. The thin films of pristine P(VDF-TrFE) and P(VDF-TrFE)/GO were called P and P-GO, respectively. Similarly, the thin films with different rGO samples were named P-rGO-SBH, PrGO-Hyd, P-rGO-AA-2h, P-rGO-AA-1d, P-rGO-AA-2d, P-rGO-AA-3d, P-rGO-HI-1, P-rGO-HI-2, P-rGO-HI-3, and P-rGO-HI-4, respectively. Figure 1a,b illustrate the processes for reduction and LS thin film preparation.

### 2.5. Characterization of r-GO and Nanocomposite Thin Film

X-ray photoelectron spectroscopy (XPS, Versaprobe II, ULVAC PHI, Tokyo, Japan) was employed to analyze surface elements and functional groups of the rGO samples. The XPS was equipped with two X-ray sources of monochromatic A1 K-alpha and a He capillary discharge light source. The photoelectrons were collected at a take-off angle of 90°. A pass energy of 187.85 eV was used to acquire high-resolution spectra of the core level regions, such as O 1s, C 1s, and the wide scan spanning an energy range of 0 to 1200 eV. The separation distance between the probes was set at 1.27 mm. Throughout the measurements, the thickness and area of the samples were maintained for consistent results at 0.2 cm and 3.14 cm^2^, respectively. Functional group analysis of the thin films was also conducted by using Fourier transform infrared (FT-IR) spectroscopy (Nicolet iS50, Thermo Fisher Scientific, Waltham, MA, USA) in the 500–4000 cm^−1^ range. For the measurement of the crystalline structure of the thin films, X-ray diffraction (XRD, D/Max2500) was employed with the parameters of an X-ray generator of 30 kV and 30 mA, scan speed of 1.00 °/min, step width of 0.02°, and angle range of 10°–30°. For the measurement of electrical conductivity of the GO and rGO samples, a four-probe electrical conductivity tester (Ossila, Sheffield, UK, T2001A2) was used, and it offers voltage and current ranges of 0.1 mV to 10 V and 10 nA to 100 mA, respectively. The pyroelectric current of PyNG was measured by a source meter (Keithley, Cleveland, OH, USA, 2400).

### 2.6. Fabrication of PyNG and Constitution of Peltier System

**Figure 2:**
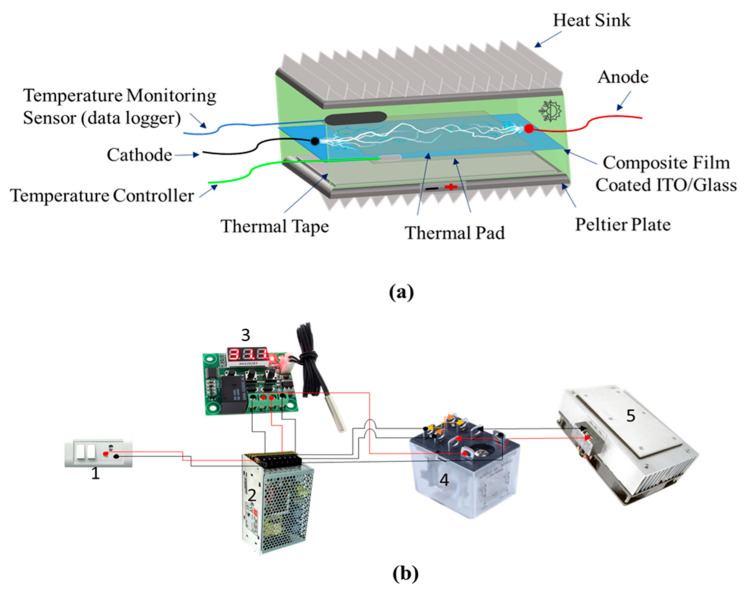
Automated Peltier system used in this work: (**a**) detailed structure of the Peltier plate and (**b**) connection among its constituents (electric switchboard (1), power supply (2), temperature controller (3), DPDT relay (4), and Peltier plate (5)).

To fabricate a PyNG, the LS film-coated area (approximately 5.5 cm × 2.5 cm) of two ITO-glasses was assembled to touch each other through the coating area, with the uncoated areas functioning as electrodes connected through alligators. A nonconductive adhesive tape was wrapped tightly around the PyNG device to prevent any gap between the ITO-glasses during the measurements. Thermal pads were covered on both sides of the PyNG to ensure equal heat transfer to the entire device. The PyNG device was then located between two Peltier plates (CPN, 12V DC, 180 W) with heat sinks on the top and bottom for heat dissipation. The Peltier plates were in direct contact with the PyNG device surface to direct heat transfer. To briefly explain the key components of the Peltier system and their operation principles, real-time temperature on the PyNG surface was recorded by a temperature monitoring sensor (Datalogger 306, Center Technology Corp., New Taipei City, Taiwan), and heating and cooling temperatures were precisely (within ±0.1 °C) controlled by a digital thermostat temperature controller (TCE, W1209) attached to a double pole double throw (DPDT) relay (MJS, R25) and a DC power supply (Smun, S-600-24, Zhejian, China). In addition, automation of the Peltier system was facilitated by a relay switch. For additional thermal insulation, cotton wool was inserted into the gaps between the Peltier plates. Figure 1c and Figure 2a,b show the PyNG of a sandwich-like structure and the key components of the Peltier system used in this work.

## 3. Results and Discussion

### 3.1. XPS Analysis

**Figure 3:**
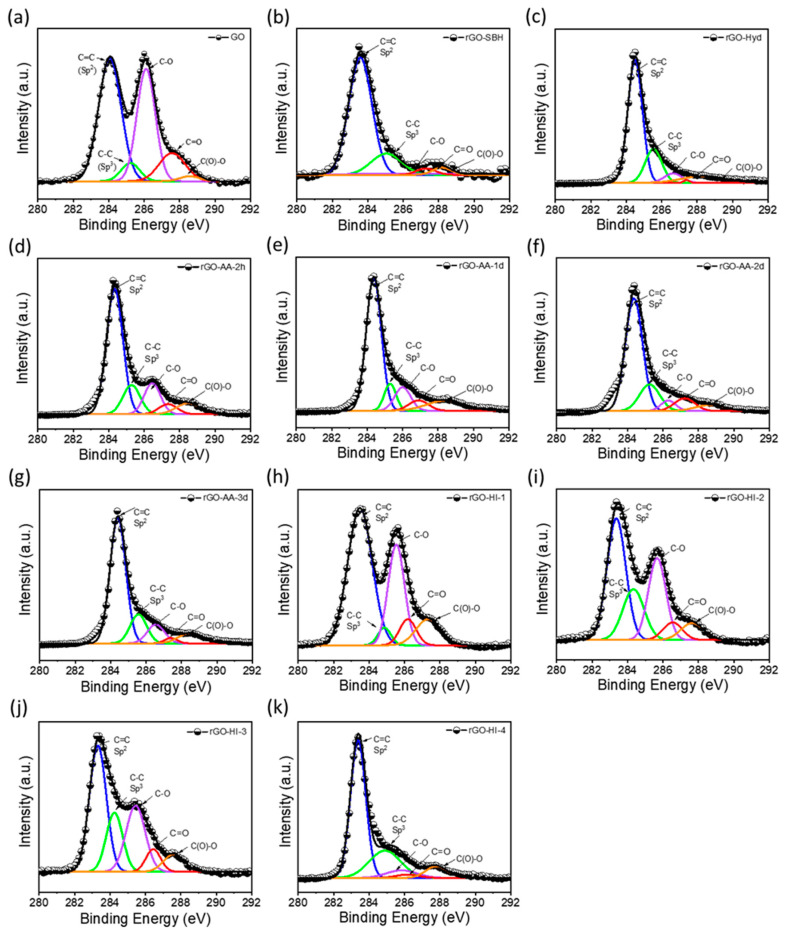
Deconvoluted C 1s spectra for (**a**) GO, (**b**) rGO-SBH, (**c**) rGO-Hyd, (**d**) rGO-AA-2h, (**e**) rGO-AA-1d, (**f**) rGO-AA-2d, (**g**) rGO-AA-3d, (**h**) rGO-HI-1, (**i**) rGO-HI-2, (**j**) rGO-HI-3, and (**k**) rGO-HI-4.

X-ray photoelectron spectroscopy (XPS) was employed to detect the removal of oxygen functional groups (OFGs) at the GO surface when GO was reduced to rGO with different reducing agents. Figure 3 shows C 1s peaks in the resulting XPS spectra of GO and rGO samples, which elucidates the variation in OFGs. The C 1s peak observed in the XPS spectra of GO prepared according to the Hummers’ method exhibits five distinct components: C=C bonding (sp^2^) at 283.20 eV, C–C bonding (sp^3^) at 284.99 eV, C–O bonding (epoxy/hydroxyl) at 285.66 eV, C=O bonding (carbonyl) at 286.29 eV, and O–C=O bonding (carboxyl) at 287.67 eV. All the rGO samples show significant intensities and peak areas corresponding to C–C and C=C peak components regardless of reducing agents, which indicates C–C and C=C bonding enrichment in the rGO samples. As shown in the figures (d) to (g) and (h) to (k), a significant decrease in intensity and area of the C–O peak component was observed with a higher concentration of HI and a longer time of AA reduction, which indicates more removal of epoxy/hydroxyl groups due to a higher degree of reduction. However, it is noted that the C–O peak component is not completely removed, even after the reduction with the highest concentration of HI for a long time. The C=O and O–C=O peak components also showed similar trends of a distinct decrease in intensity and area upon reduction, which indicates partial removal of carbonyl and carboxyl groups, respectively. Table 1 summarizes detailed information on the deconvoluted peaks and C/O ratio in the C 1s peaks for the GO and rGO samples.

As shown in Table 1, several distinct trends were identified in the reduction processes. First, the OFG ratio in percentage for the GO was 46.8% (C/O ratio 2.1), and it decreased (C/O ratio increased) upon reduction. The reference samples of rGO-SBH and rGO-Hyd yielded the same value of OFG ratio, 24.2% (C/O ratio 4.1), indicating the reducing agents of NaBH_4_ and hydrazine removed approximately half of the OFGs from the GO surface. For the rGO-HI-1, rGO-HI-2, rGO-HI-3, and rGO-HI-4, the OFG ratio decreased to approximately half from 33.7% to 16.2% (C/O ratio increased approximately two times) as the HI concentration increased ten times from 2.32 g to 23.2 g. For the rGO-AA samples, the reaction time longer than 2 h yielded significantly reduced rGO samples with an OFG ratio lower than approximately 27.2% (C/O ratio larger than 3.7). Although the eco-friendly reducing agent AA was known to have a mild reducing power, a reduction time of more than 1 day at 65 °C resulted in a significant reduction of GO, which is approximately the same results as those of a high HI concentration. In addition, the content of C=C (sp^2^) increased with increasing HI concentration and AA reduction time, which indicated that the rGO surface was changed to a more graphite-like one [11,12].

The iodide ion (I^−^) from HI is a strong nucleophile, and thus, it reacts with the OFGs on the GO substrate via the S_N_1 or S_N_2, depending on the substrate. Therefore, the I^−^ was expected to serve as an excellent reducing agent to remove the abundant OFGs, such as hydroxyl, epoxy, and carbonyl groups. In their study of GO reduction using HI and acetic acid, Lee and coworkers obtained rGO with a very high C/O ratio and electrical conductivity, and they suggested that the possible mechanisms of GO reduction by HI include iodination of alcohol, cleavage of ether, reduction of aromatic iodides, and partial reduction of the carbonyl functionalities [34,35]. On the other hand, a typical mechanism of GO reduction by AA was explained as a three-step procedure, including the S_N_2 attack of the oxyanion of AA (C_6_H_7_O_6_^−^) formed as the result of the dissociation of two protons from the AA, the intermediate formation, and the formation of rGO and dehydroascorbic acid [34,36].

### 3.2. FT-IR Analysis

**Figure 4:**
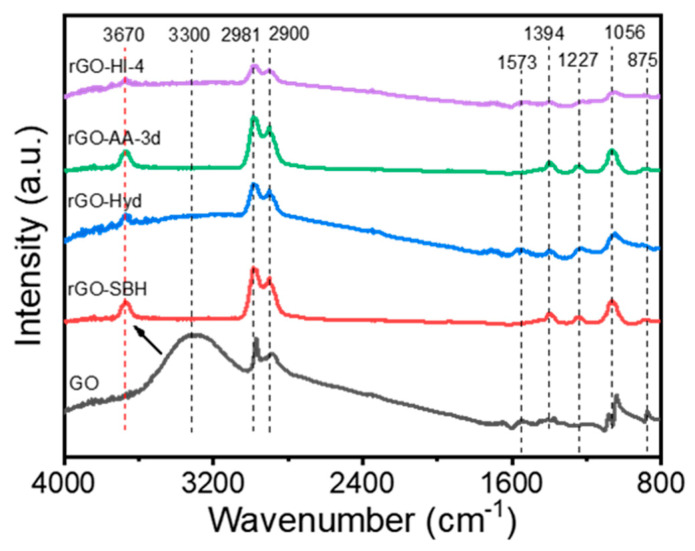
FT-IR spectra for the GO and rGO samples in the range of 500–4000 cm^−1^.

Fourier transform infrared spectroscopy (FT-IR) was employed to obtain additional information on the variation in functional groups in the reduction processes. Figure 4 shows the comparison of FT-IR spectra for the GO and rGO samples to identify changes in the intensity of the absorption peaks corresponding to specific functional groups as summarized in Table 2. As shown in Table 2, the peaks at 897, 1056, 1227, 1394, 1573, 3300, and 3670 cm^−1^ were observed for the GO and rGO samples, and they indicated the C–O stretching in epoxy, the C–O–C stretching in alkoxy, the C–O stretching in epoxy, the C=O stretching in carbonyl, and the C=C skeletal stretching, respectively [22,26,36,37,38]. When the GO samples were reduced by the reducing agents, such as SBH, Hyd, AA, and HI, the peaks at 897, 1227, and 3300 cm^−1^ almost disappeared, while those at 1056 and 1394 cm^−1^ were reduced in intensity. These results indicate that the OFGs, including epoxy, alkoxy, and carbonyl groups, disappeared or reduced upon the reduction. However, the peak at 1573 cm^−1^ was maintained with some insignificant change. In addition, the OH broad peak at 3300 cm^−1^ denoting alcohol disappeared, whereas that at 3670 cm^−1^ representing phenols appeared and reduced with more reduction (the black arrow indicates the transfer of the OH peak). These results implied that the rGO has a more aromatic structure upon the reduction and its oxygen content decreases with higher reduction.

### 3.3. XRD Analysis

**Figure 5:**
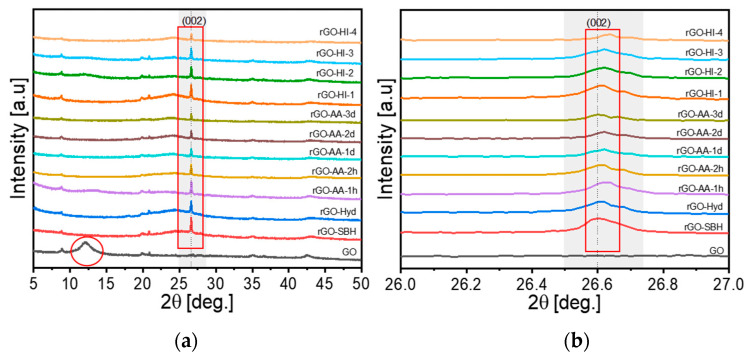
XRD patterns of the GO and rGO samples in the range of (**a**) 5–50° and (**b**) 25–28°.

X-ray diffraction (XRD) patterns of the thin films of GO and rGO were investigated to collect additional information on the change in crystallography in the reduction processes. Figure 5a,b present the XRD patterns, and Table 3 shows the diffraction peak (2θ) along (002) and the layer-to-layer distance (d-spacing) for the films of GO and rGO. The film of synthesized GO gave a diffraction peak at 12.14° with a d-spacing of 0.729 nm, which is larger than the reported d-spacing value (0.337 nm) of pristine graphite in the literature [22], which is presumably due to the intercalated oxygen functionalities and water molecules between the layers [36]. The diffraction peak shifted dramatically from 12.14° to 26.60° upon NaBH_4_ reduction as shown by the red circle and boxes in Figure 5, and it slightly moved from 26.59° to 26.64° as the reduction time increased from 2 h to 3 d upon AA reduction. These peak shifts towards higher 2θ values (12.14–26.64°) can be attributed to the reduction-induced structural rearrangements within the graphene lattice, i.e., the reduction removed OFGs leading to the reformation of sp^2^ C–C bonds and the restoration of the graphitic structure [22,30].

### 3.4. Electrical Conductivity

**Figure 6:**
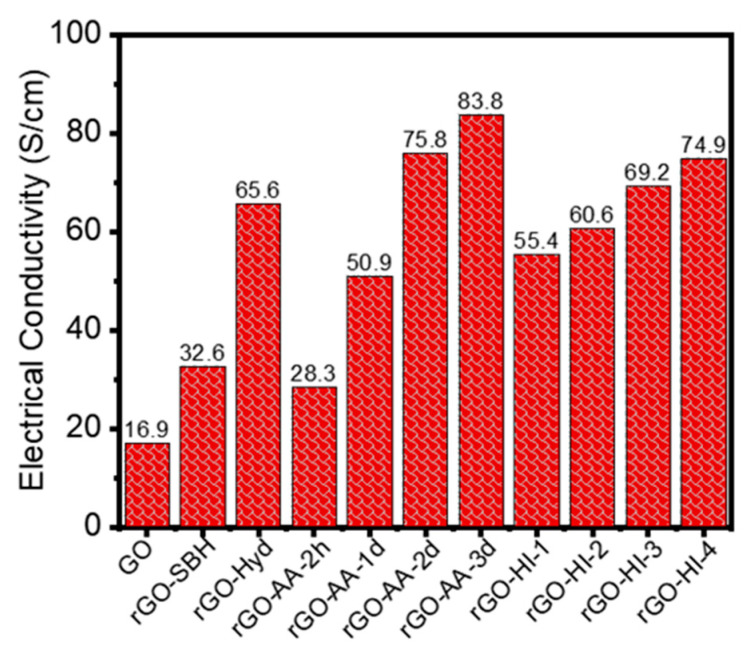
Electrical conductivity of the GO and rGO samples.

Electrical conductivity values of the GO and rGO samples were measured with a four-probe electrical conductivity tester. As shown in Figure 6, they were measured to be 16.9, 32.6, and 65.6 S/cm for GO, rGO-SBH, and rGO-Hyd, respectively. For the rGO samples prepared with AA, the electrical conductivity increased more than three times from 28.3 to 83.8 S/cm as the reduction time increased from 2 h to 3 d. Similarly, for those reduced with HI, the measured values increased from 55.4 to 74.9 S/cm with the increasing concentration of HI from 2.32 to 23.3 g. Notably, the rGO-AA-2d yielded the highest electrical conductivity value, which was somewhat unexpected because the AA has been known to be mild in reduction power. These results imply that even a weak reducing agent like the AA can produce rGO with high electrical conductivity if the reduction time is long enough at elevated temperatures.

The electrical conduction in GO and rGO sheets is governed by electron transport properties, and the OFGs at the GO and rGO surfaces serve as structural defects hampering electron transport. Therefore, an increase in electrical conductivity for the rGO upon higher reduction can be primarily attributed to the removal of OFGs. In other words, the surface OFGs, such as hydroxyl (OH), epoxy (C–O–C), and carbonyl (C=O) groups, are known to serve as defects disrupting the sp^2^ carbon network in GO. The decrease in the number of those defects by the reduction process leads to an increase in the sp^2^ carbon fraction and, thus, conjugate π-electron density, which promotes electron transport and, thus, the conductivity of the rGO [11,12,39].

### 3.5. Pyroelectric Nanogenerator Performance

#### 3.5.1. Effect of rGO Reduction

**Figure 7:**
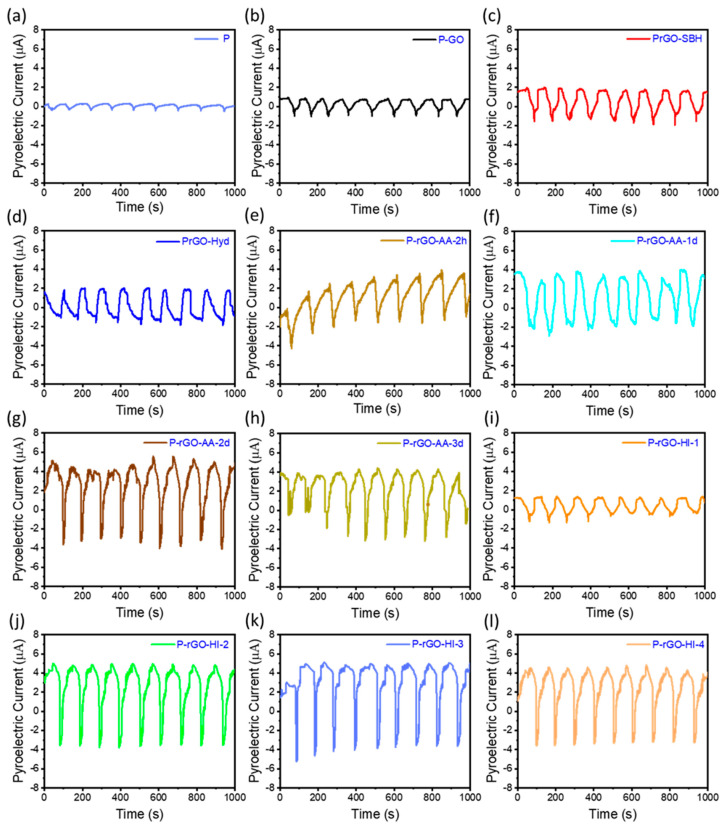
Pyroelectric current (*i*) profiles with ΔT = 20 °C for the PyNGs of (**a**) P, (**b**) P-GO, (**c**) P-rGO-SBH, (**d**) P-rGO-Hyd, (**e**) P-rGO-AA-2h, (**f**) P-rGO-AA-1d, (**g**) P-rGO-AA-2d, (**h**) P-rGO-AA-3d, (**i**) P-rGO-HI-1, (**j**) P-rGO-HI-2, (**k**) P-rGO-HI-3, and (**l**) P-rGO-HI-4.

The energy harvesting performance of PyNG was evaluated by measuring its short-circuit pyroelectric current (i) with the heating and cooling cycles in the range of 20 and 40 °C (Δ*T* = 20 °C). Figure 7 shows the i profiles for the twelve PyNGs of thin films containing 4 wt% of GO and rGO in the polymer matrix. Each thin film showed an i profile of a specific magnitude at the same time with the same temperature oscillation.

**Figure 8:**
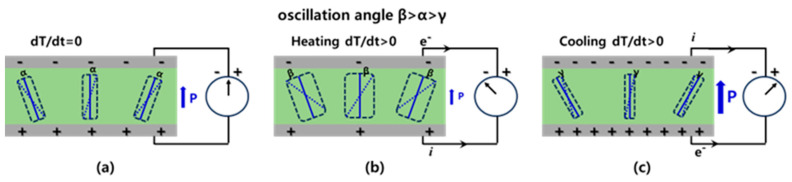
Schematic illustration of the operation mechanism of the PyNG of P(VDF-TrFE)/rGO thin film: dipole oscillation and pyroelectric current when (**a**) *dT/dt* = 0, (**b**) *dT/dt* > 0, and (**c**) *dT/dt* < 0.

The operation mechanism of the PyNG of the P(VDF-TrFE)/rGO thin film can be explained in terms of the change in the spontaneous polarization of the nanocomposite film as shown in Figure 8. When the temperature is constant, i.e., *dT/dt* = 0 (Figure 8a), the spontaneous polarization of electric dipoles is constant with a medium oscillation angle. When the PyNG is heated up by the Peltier plate as shown in Figure 2a, i.e., *dT/dt* > 0 (Figure 8b), the electrical dipoles oscillate in a wider range yielding the electron flow from the anode to the cathode. On the contrary, when the PyNG is cooled down to the original temperature, *dT/dt* < 0 (Figure 8c), the electrical dipoles oscillate at a narrower angle generating a current opposite to that in Figure 8b. As such, the oscillating pyroelectric current curves are generated per the temperature fluctuation without any significant time delay.

**Figure 9:**
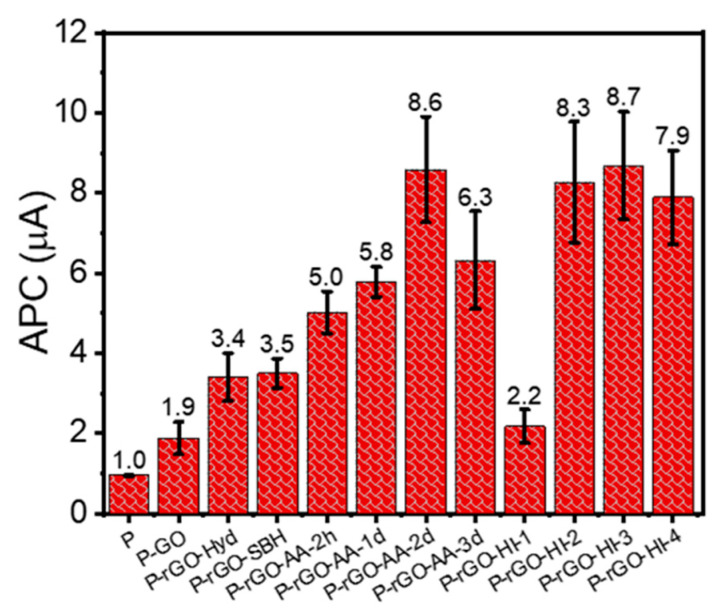
Average peak-to-peak pyroelectric current (APC) values for the GO and rGO samples.

Figure 9 shows the average peak-to-peak pyroelectric current (APC) values with error bars for the twelve PyNGs. The PyNG of the P (pristine P(VDF-TrFE)) thin film yielded the APC value of 0.97 μA. The PyNGs of P-GO, P-rGO-SBH, P-rGO-Hyd, and P-rGO-HI-1 showed slightly higher APC values of 1.9, 3.4, and 3.5 μA than that of pristine P(VDF-TrFE), respectively. Meanwhile, the PyNGs of the P-rGO-HI-2, P-rGO-HI-3, and P-rGO-HI-4 films yielded relatively higher APC values of 8.3, 8.7, and 7.9 mA, respectively. These results are presumably due to the higher electrical conductivity of the dispersed phase of rGO due to the larger magnitude of reduction. In other words, NaBH_4_, hydrazine, and 55% HI 2.32 g were not strongly reducing enough to yield higher pyroelectricity, which is partially consistent with the electrical conductivity results. In addition, the P-rGO-AA-2h, P-rGO-AA-1d, P-rGO-AA-2d, and P-rGO-AA-3d films presented much higher APC values of 5.0, 5.8, 8.6, and 6.3 mA. Importantly, the APC value for P-rGO-AA-2d was the highest, which was almost the same as that for P-rGO-HI-3, due to its highest electrical conductivity. Consequently, the APC values for the twelve thin films were in the order of P-rGO-HI-3 > P-rGO-AA-2d > P-rGO-HI-2 > P-rGO-HI-4 > P-rGO-AA-3d > P-rGO-AA-1d > P-rGO-AA-2h > P-rGO-SBH ≈ P-rGO-Hyd > P-rGO-HI-1 ≈ P-GO > P.

There are three noticeable trends in the APC results: (i) All the APC values for the thin films containing GO and rGO are larger than that for the pristine P(VDF-TrFE) thin film; (ii) All the thin films having rGO show larger APC values than that for the film with GO; (iii) For the films having rGO prepared by the HI and AA reduction, the APC value increases with increasing electrical conductivity of rGO, except the thin films of the highest electrical conductivity. These trends can be effectively explained by discussing pyroelectric properties in terms of the Maxwell–Wagner effect in a two-phase system [40,41,42,43].

In principle, the pyroelectric current, i, essentially depends on the rate of temperature fluctuation (*dT*/*dt*), the active surface area of the electrode (*A*), and the pyroelectric coefficient (*p*), which is defined as spontaneous polarization (*P_S_*) per unit temperature, according to the following Equation (1):

(1) $$i=\frac{dQ}{dt}=A\frac{dPs}{dt}=A\frac{dPs}{dT(t)}\frac{dT(t)}{dt}=A\;p\frac{dT(t)}{dt}$$

where *Q* denotes the induced charges. The pyroelectric coefficient, *p*, is one of the most important factors influencing the pyroelectric performance of thin films [43].

In this work, the nanocomposite film was composed of two phases, i.e., phase 2 of non-ferroelectric GO or rGO particles was dispersed in phase 1 of the P(VDF-TrFE) matrix. In this kind of two-phase system, the total pyroelectric and ferroelectric properties are known to be described with a combination of parallel and series models, and they can be controlled by the conductivity, *σ*_2_, of the dispersed phase (phase 2) [40,41,42,43]. The dielectric displacement, *D*, and the electric field, *E*, in the composite film are linearly correlated as follows:

(2) $$D=D_{0}+\varepsilon E$$

where *D*_0_ and *ε* denote the average dielectric displacement at *E* = 0 and the dielectric constant of the composite film, respectively. Similarly, the dielectric displacement for phases 1 and 2, *D*_1_ and *D*_2_, can be expressed as follows:

(3) $$D_{1}=\varepsilon_{1}E_{1}+p_{1}$$

(4) $$D_{2}=\varepsilon_{2}E_{2}+p_{2}\approx \varepsilon_{2}E_{2}$$

where *E*_1_ and *E*_2_ are local electric fields for the phases, respectively, and *p*_1_ and *p*_2_ are pyroelectric coefficients for phases 1 and 2, respectively. Here, *p*_2_ = 0 because phase 2 is non-ferroelectric. With a given volume fraction, *Φ* of phase 2, the average electric field, *E*, and the average dielectric displacement, *D*, can be expressed as follows:

(5) $$E=\left(1-\Phi \right)E_{1}+\Phi E_{2}$$

(6) $$D=(1-\Phi )D_{1}+\Phi D_{2}$$

Considering the principle of pyroelectricity, total pyroelectric current, *i*, in the composite film can be expressed by the following equation:

(7) $$i=\frac{\partial D_{1}}{\partial t}+\sigma_{1}E_{1}=\frac{\partial D_{2}}{\partial t}+\sigma_{2}E_{2}$$

For the two-phase system in this study, the electrical conductivity, *σ*_2_, and the dielectric constant, *ε*_2_, of phase 2 (rGO) have been known to be larger than *σ*_1_ and *ε*_1_ of phase 1 (P(VDF-TrFE)). From the results in Figure 6, *σ*_2_ = 16.9 to 83.8 S/cm for rGO. From the literature, *ε*_2_ = 1130 for rGO [44], *σ*_1_ = 1.0 × 10^−4^ S/cm, and *ε*_1_ = 36 for P(VDF-TrFE) [45]. The following equation is obtained by applying Equations (3) and (4) and the relationship of *σ*_2_ ≫ *σ*_1_ to Equation (7):

(8) $$\varepsilon_{1}\frac{\partial E_{1}}{\partial t}+\frac{\partial p_{1}}{\partial t}=\varepsilon_{2}\frac{\partial E_{2}}{\partial t}+\sigma_{2}E_{2}$$

By the application of the expression for *E*_2_ obtained from Equation (5) to Equation (8) and rearrangement, the following equation is obtained:

(9) $$\frac{\partial p_{1}}{\partial t}=\varepsilon_{2}[\frac{1}{\Phi }\frac{\partial E}{\partial t}+\left(\frac{\Phi -1}{\Phi }\right)\frac{\partial E_{1}}{\partial t}]+\frac{\sigma_{2}}{\Phi }E+(\frac{\Phi -1}{\Phi })\sigma_{2}E_{2}-\varepsilon_{1}\frac{\partial E_{1}}{\partial t}$$

According to the Maxwell-Wagner effect, space charge layers are formed at the interface between the P(VDF-TrFE) matrix and the included rGO when the strong electric field is applied during the poling. These layers increase the bound charges on the electrodes in phase with the total applied electric field *E*, which increases the dielectric constant of the composite film. In this environment, *E*_1_ reaches a critical electric field, *E_c_*, and the spontaneous polarization of the polymer matrix happens when *E*_1_ remains constant. When the following conditions hold, Equation (9) becomes the following simplified equation by the integration:

(10) $$\frac{\partial E_{1}}{\partial t}=0\;\;\;\&\;\;\;E_{1}=E_{C}$$

(11) $$p_{1}=\varepsilon_{2}\left(\frac{E}{\Phi }\right)+\frac{\sigma_{2}}{\Phi }[E+\left(\Phi -1\right)E_{1}]t$$

Therefore, it is concluded that the pyroelectric coefficient in the polymer matrix, *p*_1_, is proportional to the electrical conductivity, *σ*_2_, and the dielectric constant, *ε*_2_, of phase 2 (rGO). In addition, it is known that rGO has a higher dielectric constant than GO [44]. Therefore, this insightful analysis using theoretical interpretation well explains the second and third trends of APC values in Figure 9. In other words, the *p* value of the P(VDF-TrFE)/rGO nanocomposite film increases by the combination effect of increasing electrical conductivity and dielectric constant of rGO upon more reduction.

In addition, the local field coefficients *L_E_*_1_ and *L_E_*_2_ for phases 1 and 2 can be expressed as the following equations [42]:

(12) $$L_{E_{1}}=\frac{E_{1}}{E}=\left(\frac{1}{1-\Phi }\right)\left(\frac{\varepsilon_{2}-\varepsilon }{\varepsilon_{2}-\varepsilon_{1}}\right)$$

(13) $$L_{E_{2}}=\frac{E_{2}}{E}=\left(\frac{1}{\Phi }\right)\left(\frac{\varepsilon_{1}-\varepsilon }{\varepsilon_{1}-\varepsilon_{2}}\right)$$

For the two-phase system in this study, the relationship of *L_E_*_1_ ≫ *L_E_*_2_ holds from Equations (12) and (13) because *ε*_2_ ≫ *ε*_1_, and thus, the local electric field applied on phase 1 (PVDF-TrFE) is much higher than that on phase 2 (rGO). As a result, the P(VDF-TrFE) matrix can be easily poled, and thus, the required poling electric field is reduced. This can explain why the pyroelectric current of the nanocomposite film is higher than that of the pristine P(VDF-TrFE) film [41,42]. However, too high conductivity of the composite film can induce dissipation and deterioration of pyroelectric and ferroelectric properties due to current leakage and charge dissipation [41,42,46]. This analysis well explains the slight reduction in pyroelectric current in the thin films of P-rGO-AA-3d and P-rGO-HI-4 with the highest electrical conductivity.

#### 3.5.2. Effect of Temperature

**Figure 10:**
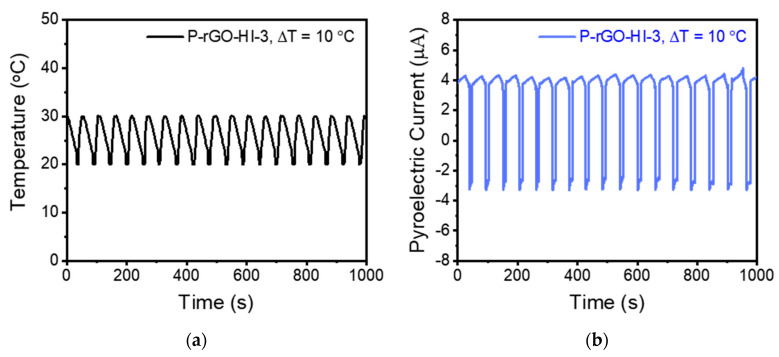
(**a**) Temperature profile and (**b**) corresponding *i* profile for the P-rGO-HI-3 thin film with Δ*T* = 10 °C.

**Figure 11:**
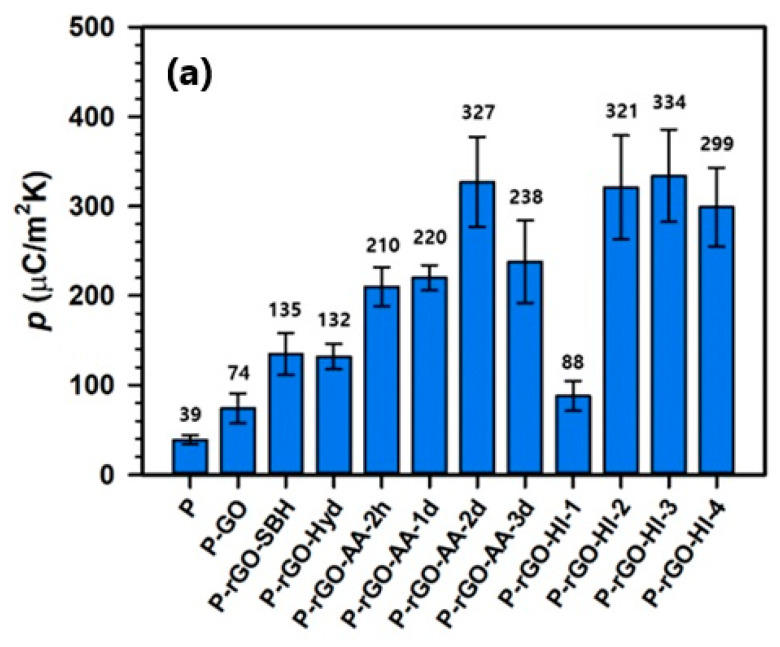

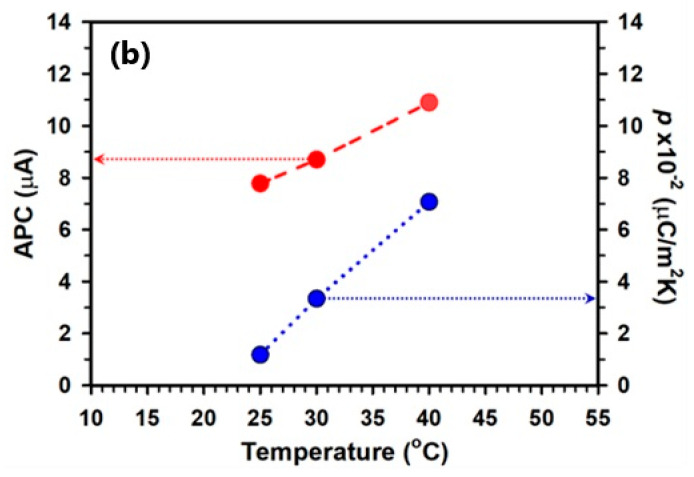
(**a**) Comparison of all the *p* values for the twelve thin films and (**b**) temperature dependence of the APC and *p* values (the red arrow and the blue arrow indicate corresponding APC and *p* values, respectively).

From the *i* profiles in Figure 9 and Equation (1), the *p* values for the thin films can be readily evaluated. Figure 10a,b show the temperature and the representative *i* profiles for P-rGO-HI-3, and Figure 11a compares all the evaluated *p* values for the twelve thin films, which showed a similar trend to that of the APC value. As a reference, the *p* value for the pristine P(VDF-TrFE) film was evaluated as 39 μC/m^2^∙K, which is comparable to those (31–55 μC/m^2^∙K) in the literature [1,47]. Notably, P-rGO-AA-2d and P-rGO-HI-3 yielded the highest *p* values of 327 and 334 μC/(m^2^∙K), respectively, which are much larger than that for the pristine P(VDF-TrFE) film. For the P-rGO-HI-3 film, the *i* measurement was repeated in two different temperature ranges of 20 to 30 °C and 20 to 60 °C. By the same method as before, the APC and the *p* values were evaluated, and the results were compared with those in the range of 20 to 40 °C in Figure 11b (the data were plotted at the middle points in the temperature ranges, i.e., at 25 °C for 20 to 30 °C, at 30 °C for 20 to 40 °C, and at 40 °C for 20 to 60 °C).

The APC values of 7.78, 8.70, and 10.9 mA and the *p* values of 118, 334, and 707 μC/(m^2^∙K) were obtained for three different temperature ranges of 20–30 °C, 20–40 °C, and 20–60 °C, respectively. Both the APC value and the *p* values gradually increased with increasing temperature range. This type of temperature dependence of the *p* value for a ferroelectric polymer matrix like PVDF has been experimentally observed and well explained in terms of the change in polymer semicrystalline structure upon temperature change [2,48,49]. To describe the details, the *p* value of a ferroelectric polymer like PVDF gradually increases through three transitions among four regions of different slopes when it is heated from −100 to 80 °C: The first transition is at the glass transition (−42 °C for PVDF) of the polymer; the second transition is at approximately 18 °C, where loops in the folded polymer chains are presumably loosened and the amorphous phase decoupled from crystallites becomes free; and the third transition is at approximately 47 °C, where conformation at the lamellae surface is presumably reorganized, which is called the α-process [49]. Therefore, the *p* value in this work varied presumably due to the conformational reorganization of polymer chains in the region of the α-process because all the temperature ranges were between 20 and 60 °C. For the temperature range of 20 to 40 °C, the power density was calculated to be 3.85 mW/cm^2^ with APC and *p* values of 8.7 mA and 334 μC/m^2^∙K, respectively. As shown in Table 4, these results were found to be superior to those in other studies regarding the pyroelectric performance of a ferroelectric polymer containing carbon-based nanofillers, such as GO, rGO, and CNT, in the literature. As previously discussed, the current PyNG’s excellent performance can be ascribed to the highly ordered and uniform structure of the molecularly thin film matrix and the optimized electrical conductivity and dielectric constant of the rGO due to properly optimized reduction. Improved energy-harvesting performance of the current PyNG is expected to bring about advancement in the effectiveness of PyNG by providing critical information and making it promising for application in the area of portable microelectronics and wearable devices.

## 4. Conclusions

In summary, we fabricated various pyroelectric nanogenerators (PyNGs) comprising P(VDF-TrFE)/rGO nanocomposite thin films containing rGO prepared by different reduction methods, and we evaluated their energy-harvesting performance by measuring the pyroelectric current (*i*) and evaluating the pyroelectric coefficient (*p*) in three different temperature ranges. In the temperature range of 20 to 40 °C, the thin films containing rGO samples reduced at the HI concentration of 11.6 g and at the AA reduction time of 2 days yielded the highest average pyroelectric peak-to-peak current (APC) and pyroelectric coefficient (*p*) values. Importantly, it was found that the thin films containing more reduced rGO had higher APC and *p* values and, thus, yielded better energy-harvesting performance. These results were ascribed mainly to the higher electrical conductivity of rGO due to more reduction. However, the thin films containing rGO with too high electric conductivity produced slightly reduced performance. These optimization results were successfully explained by employing the Maxwell–Wagner effect in a two-phase ferroelectric system. The results of the energy density of 3.85 mW/cm^2^ and the *p* value of 334 μC/(m^2^∙K) for Δ*T* = 20 °C were found to be superior to those in other studies in the literature, and this excellent performance was presumably ascribed to the well-ordered and uniform structure of the nanocomposite thin film and optimized electrical conductivity of rGO by the proper amount of reduction. The improved energy-harvesting performance of the current PyNG provides useful information for the development of an effective PyNG and makes it promising for application in microelectronics including wearable devices.

## Figures and Tables

**Table 1:** Deconvoluted peaks and C/O ratio in the C 1s peaks of the XPS spectra for the GO and rGO samples.

Sample	C=C (sp^2^)	C–C (sp^3^)	C–O	C=O	O–C=O	OFG Ratio (%)	C/O Ratio
	283.20 eV	284.99 eV	285.66 eV	286.29 eV	287.67 eV		
GO	46.0	7.2	29.8	10.8	6.2	46.8	2.1
rGO-SBH	56.9	18.9	7.0	8.4	8.8	24.2	4.1
rGO-Hyd	59.4	16.4	10.4	5.7	8.1	24.2	4.1
rGO-AA-2h	50.1	22.7	14.9	4.9	7.4	27.2	3.7
rGO-AA-1d	65.3	16.8	6.0	4.4	7.5	17.9	5.6
rGO-AA-2d	70.2	12.3	5.0	3.7	8.8	17.5	5.7
rGO-AA-3d	73.9	9.6	5.6	4.9	6.0	16.5	6.1
rGO-HI-1	47.3	19.0	17.1	4.2	12.4	33.7	3.0
rGO-HI-2	50.4	17.5	13.2	7.8	11.1	32.1	3.1
rGO-HI-3	59.9	19.3	5.7	6.1	9.0	20.8	4.8
rGO-HI-4	70.1	13.8	6.1	2.2	7.9	16.2	6.2

**Table 2:** Summary of OFGs in the FT-IR spectra for the GO and rGO samples [22,26,36,37,38].

Absorption Peaks (cm^−1^)	Bond	Functional Groups	Intensity Change in rGO Spectra
897	C–O stretching	Epoxy	disappeared
1056	C–O–C stretching	Alkoxy	reduced
1227	C–O stretching	Epoxy	disappeared
1394	C=O stretching	Carbonyl	reduced
1573	C=C stretching	Aromatic	maintained
3300	O–H stretching	Alcohols	disappeared
3670	O–H stretching	Phenols	appeared but reduced

**Table 3:** Diffraction peak angle along (002) in the XRD patterns of the GO and rGO samples.

Samples	2θ [°]
GO	12.14
rGO-SBH	26.60
rGO-Hyd	26.60
rGO-AA-2h	26.59
rGO-AA-1d	26.62
rGO-AA-2d	26.64
rGO-AA-3d	26.64
rGO-HI-1	26.62
rGO-HI-2	26.62
rGO-HI-3	26.62
rGO-HI-4	26.64

**Table 4:** Pyroelectric performance comparison of other studies with PVDF film containing carbon-based nanofillers in the literature.

Material	Temperature Source	Δ*T* (K)	Power Density (mW/cm^2^)	*p* Value (μC/m^2^∙K)	Reference
PVDF/GO	Water	60	20.0	38	[50]
PVDF-GO nanofibers	Breathing	22	6.2 × 10^−4^	2.7 × 10^−2^	[51]
Au@CNT/PVDF	Sunlight	41.3	1.5	-	[52]
CNT/CNC-PVDF	Solar-thermal	11.2	0.9 × 10^−4^	-	[53]
PVDF/rGO-PEI	Solar thermal	37	2.13 × 10^−3^	-	[54]
PVDF/Ag@rGO-PEI	Solar-thermal	29	0.94	-	[55]
P(VDF-TrFE)/rGO	Automated Peltier System	20	3.85	334	This work

## Data Availability

All the data are included within the article, and the raw data are available upon request.
